# Higher Accumulation of Docosahexaenoic Acid in the Vermilion of the Human Lip than in the Skin

**DOI:** 10.3390/ijms21082807

**Published:** 2020-04-17

**Authors:** Md. Al Mamun, Shumpei Sato, Eiji Naru, Osamu Sakata, Emi Hoshikawa, Ayako Suzuki, Ariful Islam, Tomoaki Kahyo, Tomohito Sato, Takashi K. Ito, Makoto Horikawa, Reimu Fukui, Kenji Izumi, Mitsutoshi Setou

**Affiliations:** 1 Department of Cellular & Molecular Anatomy, Hamamatsu University School of Medicine, 1-20-1 Handayama, Higashi-ku, Hamamatsu, Shizuoka 431-3192, Japan; d17105@hama-med.ac.jp (M.A.M.); 07485372_old@hama-med.ac.jp (S.S.); d17103@hama-med.ac.jp (A.I.); kahyo@hama-med.ac.jp (T.K.); 07485496@hama-med.ac.jp (T.S.); itotk777@hama-med.ac.jp (T.K.I.); makotoh@hama-med.ac.jp (M.H.); reimufki@hama-med.ac.jp (R.F.); 2 Research Laboratories, KOSÉ Corporation, 1-18-4 Sakae-cho, Kita-ku, Tokyo 114-0005, Japan; e-naru@kose.co.jp (E.N.); o-sakata@kose.co.jp (O.S.); 3 Division of Biomimetics, Faculty of Dentistry & Graduate School of Medical and Dental Sciences, Niigata University, 2-5274 Gakkocho-dori, Chuo-ku, Niigata City 951-8514, Japan; hoshikawa@dent.niigata-u.ac.jp (E.H.); suzuki-a@dent.niigata-u.ac.jp (A.S.); izumik@dent.niigata-u.ac.jp (K.I.); 4 International Mass Imaging Center, Hamamatsu University School of Medicine, 1-20-1 Handayama, Higashi-ku, Hamamatsu, Shizuoka 431-3192, Japan; 5 Department of Systems Molecular Anatomy, Institute for Medical Photonics Research, Preeminent Medical Photonics Education & Research Center, 1-20-1 Handayama, Higashi-ku, Hamamatsu, Shizuoka 431-3192, Japan

**Keywords:** DESI–MSI, docosahexaenoic acid, free fatty acids, human lip, skin, vermilion

## Abstract

The vermilion of the human lip is a unique facial area because of certain distinguishing features from the adjacent tissues such as the white lip (skin) and oral mucosa. However, the distinction in terms of molecular distribution between the vermilion and skin has remained unexplored. Therefore, we aimed to map the human lip by mass spectrometry imaging to gain understanding of the free fatty acid distribution in the vermilion. The lip specimens trimmed off during cheiloplasty were analyzed using desorption electrospray ionization–mass spectrometry imaging. Distributions of two monounsaturated fatty acids and three polyunsaturated fatty acids were observed in the human lip tissue: palmitoleic acid (POA) and oleic acid (OA) and linoleic acid (LA), arachidonic acid (AA), and docosahexaenoic acid (DHA), respectively. Although POA, OA, LA, and AA were differentially distributed across the vermilion and skin, DHA showed a higher accumulation in the epithelium of the vermilion compared to that in the skin. Our results clearly demonstrated the difference in fatty acid distributions between the vermilion and skin. The highly abundant DHA in the epithelium of the vermilion may have an antioxidant role and may thus protect the lip from aging. Our findings can provide a novel strategy for treating lip disorders.

## 1. Introduction

**Figure 1:**
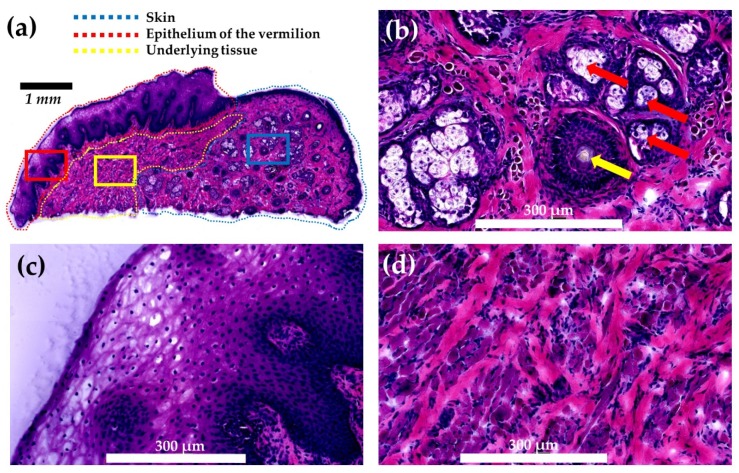
Histology of lip tissue under this study. The tissue obtained from subject 2 was stained with hematoxylin and eosin. (**a**) Whole tissue section showing the skin, epithelium of the vermilion, and its underlying tissue. Magnified view of (**b**) the skin showing the hair follicle (indicated by yellow arrow) and sebaceous glands (indicated by red arrows). Magnified view of (**c**) the epithelium of the vermilion, and its (**d**) underlying tissue. Areas encircled by blue, red, and yellow rectangles in (**a**) were used for magnification for the skin, epithelium of the vermilion, and its underlying tissue, respectively.

The lips are a unique part of the human body and play a crucial role in facial expression, phonation, sensation, mastication, physical attraction, and intimacy [1]. Each lip comprises three distinct basic parts: (i) skin, the outer part; (ii) vermilion, the transitional area between the oral mucosa and the skin; and (iii) oral mucosa, the inner part [2]. The vermilion consists of an epithelium and the underlying tissue structures (Figure 1). Compared with skin, vermilion lacks hair, sweat glands, and sebaceous glands associated with hair follicles (Figure 1) [2], and it shows poor barrier functions and water holding capacity [3,4]. The epithelium of vermilion is lightly keratinized [2], much thicker than that of the skin [5], and partly transparent to the color of the blood in the rich microvasculature of the underlying tissue [2]. Given these distinguishing histologic features, the molecular distributions contributing to these intrinsic differences are likely to be distinct between the skin and the vermilion. Therefore, modalities to visualize molecular distributions between the vermilion and the skin would be of great interest.

Free fatty acids (FFAs) play a critical role in maintaining skin homeostasis [6]. Several researchers have studied the composition [7,8,9] and distribution [10] of FFAs in the human skin and have identified a distinct profile, particularly of the epidermis. However, FFA distributions in the lip have not been studied yet. Mass spectrometry imaging (MSI) is a powerful technique that enables label-free simultaneous visualization of hundreds to thousands of molecular species from a sample surface while preserving its morphology [11]. Compared to conventional mass spectrometry (MS), such as liquid chromatography–MS, which requires extraction and large amounts of tissue, MSI can directly analyze tissue sections [11]. Previously, our group revealed the altered distribution of some phospholipid levels in several diseased tissues using matrix-assisted laser desorption ionization–MSI, a widely employed MSI technique [12,13]. Recently, desorption electrospray ionization–MSI (DESI–MSI) has emerged as an alternative tool for the detection of small molecules [14]. The advantages of DESI–MSI over MALDI–MSI include the non-necessity of sample pre-treatment and lesser invasiveness to tissue structure during measurement, such as matrix deposition [15,16]. Using DESI–MSI, our group identified several lipid biomarkers in clear cell renal carcinoma tissue [17], detected FFAs and their metabolites in thin-cap atherosclerotic plaque [18], and analyzed active components in a traditional Cuban beverage [19].

In this study, we aimed to map the human lip by DESI–MSI to gain understanding of the FFAs distribution and thus to molecularly distinguish the vermilion from the skin.

## 2. Results

### 2.1. DESI–MSI of Human Lip

**Figure 2:**
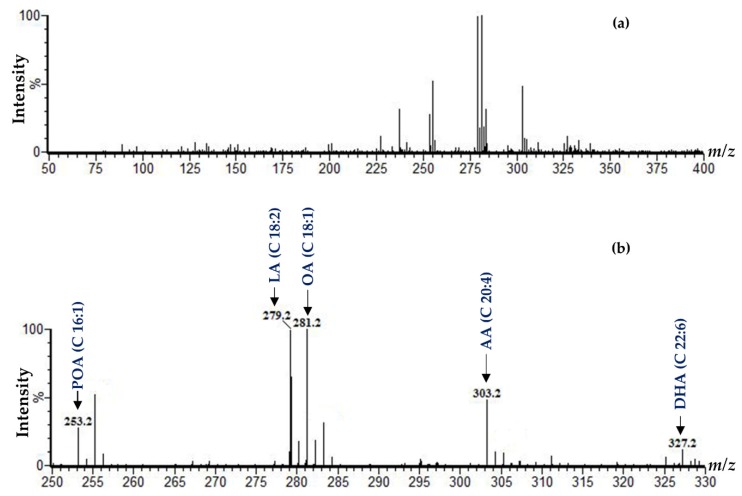
DESI–MSI spectra obtained from whole lip tissue of subject 1. (**a**) Full negative-ion mass spectrum from *m*/*z* 50−400. **(b)** Expanded view of the mass ranges from *m*/*z* 250 to 330 with molecular assignments. PA: palmitoleic acid; LA: linoleic acid; OA: oleic acid; AA: arachidonic acid; DHA: docosahexaenoic acid.

In this study, we analyzed three human lip tissues containing the skin and vermilion (epithelium of the vermilion and its underlying tissue) using DESI–MSI. The demographic data are shown in Table 1. We observed high-intensity peaks of some ions in the range of *m*/*z* 250 to 330 (Figure 2), the region that allows the detection of the most important FFAs in human and animal tissue. A total of five ions at *m*/*z* 253, 279, 281, 303, and 327 were found to be abundant in this range and were assigned as palmitoleic acid (POA), linoleic acid (LA), oleic acid (OA), arachidonic acid (AA), and docosahexaenoic acid (DHA), respectively, based on the criteria mentioned in the methods section (Table 2, Figure 2). As seen in Figure 2b, OA was detected as the most abundant monounsaturated fatty acid (MUFA) followed by POA. The relative abundance of the polyunsaturated fatty acid (PUFA) was in the order of LA > AA > DHA. Spectra extracted from the skin, epithelium of the vermilion, and underlying tissue of the vermilion of subject 1 showed the differences of abundance of the detected ions in those regions (Figure S1). 

### 2.2. Several FFAs are Differentially Distributed Across the Vermilion and Skin

**Figure 3:**
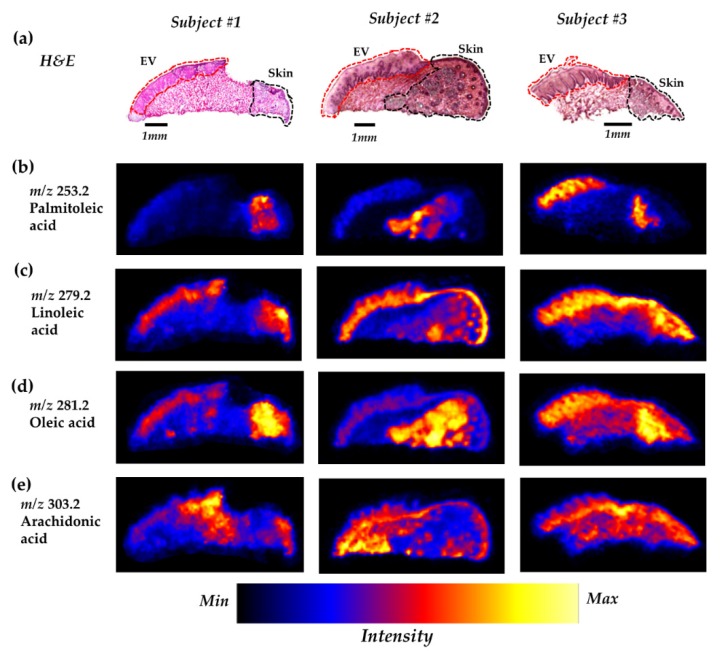
Prominent distributions of some free fatty acids across the vermilion and skin of the human lip. (**a**) Hematoxylin and eosin (H&E) images of lip section. Representative DESI–MSI ion images for (**b**) POA, (**c**) LA, (**d**) OA, and (**e**) AA. The area below the EV (not indicated in the H&E images) is the underlying tissue of the vermilion. EV: epithelium of the vermilion.

We observed a different distribution pattern between the MUFAs and PUFAs (two out of three PUFAs detected), whereas the pattern was comparable within both MUFAs and PUFAs (Figure 3). 

POA and OA had a unique distribution localized in the underlying tissue of skin in subject 1 and 2. However, in subject 3, they were present notably in the epithelium of the vermilion as well (Figure 3b,d). OA also showed a distribution in the epithelium of the vermilion of subject 1 as well as in the underlying tissue of the vermilion of subject 3 (Figure 3d).

Distributions of LA and AA are seen in both skin and epithelium of the vermilion (Figure 3c,e), whereas the distribution pattern of LA was almost similar in all three samples (Figure 3c); the distribution of AA varied between samples (Figure 3e).

### 2.3. DHA is Highly Distributed in the Epithelium of the Vermilion

**Figure 4:**
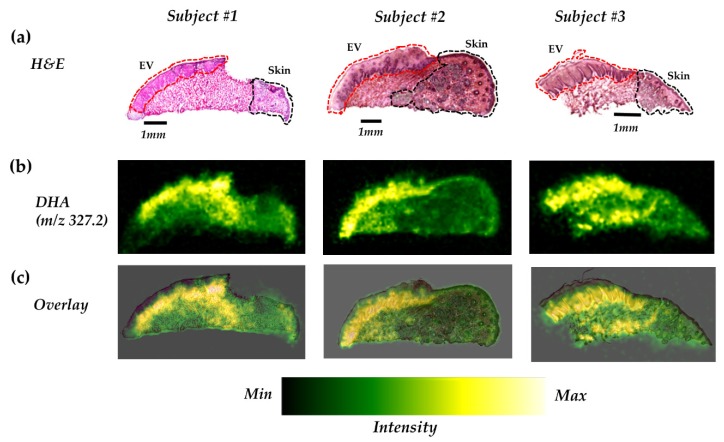
DHA is highly distributed in the epithelium of the vermilion (EV). (**a**) H&E images of lip sections, (**b**) representative DESI–MSI ion images for DHA of the human lip in negative ion mode, and (**c**) transparent overlaid images for DHA and H&E. The skin and EV areas are indicated by black and red lines, respectively, in the H&E images. The area below the EV (not indicated) is the underlying tissue of the vermilion.

Although DHA had the lowest abundance among the five FFAs detected in the present study, unexpectedly, a distinctively higher distribution of DHA was found in the vermilion, particularly in the epithelium of the vermilion, in comparison to the adjacent epidermis (skin) (Figure 4). To identify the precise distribution of DHA in the epithelium of the vermilion, we overlaid the ion images of each subject with the corresponding hematoxylin and eosin (H&E) images. DHA was found to be distinctively rich in the viable layer of the lower epithelium of the vermilion, but not in the superficial layer. In subjects 1 and 3, DHA was localized notably in some portions of the underlying tissue as well.

## 3. Discussion

The human lip has three distinct portions anatomically: the (i) skin, (ii) vermilion, and (iii) oral mucosa [2]. To the best of our knowledge, for the first time, we have mapped the human lip with a goal to distinguish the vermilion from the skin in terms of fatty acid distributions. 

We used highly sensitive DESI–MSI and observed the prominent distributions of two MUFAs, POA and OA, and three PUFAs, LA, AA, and DHA, in lip tissues of infants (Figure 3 and Figure 4). It is well known that fatty acids display a highly active metabolism in the skin [22] and play a critical role in maintaining the functions of the cutaneous cells [23]. Our results are consistent with those of the previous studies [5,22] in which the 18-carbon fatty acid OA was identified as the most abundant fatty acid and LA as the most abundant PUFA, followed by AA and DHA, in the skin area.

Of the five FFAs detected in our study, DHA had a distinct distribution in the epithelium of the vermilion (Figure 4), which made a remarkable difference between the vermilion and the skin. DHA is an important omega-3 PUFA in the human body and is well known for its multiple beneficiary effects [20,24,25]. In the vermilion, DHA may play a role as an antioxidant and thus contribute to the anti-aging of the lip.

Although the role of DHA in lip health has not been studied well, it is clear that omega-3 PUFAs, including DHA, contribute to the structural integrity of the skin and metabolize to bioactive lipid species to mediate anti-inflammatory reactions in many tissues, including the skin [26,27]. DHA has been studied extensively for its beneficial effects in several diseases including skin disorders [23]. DHA pretreatment has been shown to inhibit ultraviolet-induced inflammation in mouse skin by several mechanisms: (i) decreasing cyclooxygenase-2, nicotinamide adenine dinucleotide phosphate: oxidase-4 by blocking MSK1 signaling [28] and (ii) increasing Nrf2 activation and upregulating cytoprotective genes [29]. In one investigation, Arantes et al. found that topical DHA was capable of accelerating wound healing in a rat model of skin wounds [30]. In addition, DHA inhibits hyperpigmentation [31] and prevents skin cancer development when given in combination with anticancer drugs [32]. Therefore, we speculate that the high abundance of DHA may protect the lip as the vermilion is constantly exposed to external stimuli, and it could be associated with the rapid turnover of the vermilion surface.

The human lip is one of the major targets of cosmetics. Therefore, it is important to understand the molecular profile specific to the human lip to discover the intrinsic ingredients for superior lip cosmetics. From our finding, it is relevant to speculate that DHA could be an intrinsic and efficient ingredient of lip cosmetics with minimal side effects.

The major essential fatty acid, LA, cannot be synthesized in vivo and must be supplemented through diet, since the mammalian system is incapable of inserting double bonds beyond the n-9 position [23]. An important function of LA is to maintain the barrier function of the stratum corneum by producing ceramides [23,33]. LA also acts as a precursor of AA [23], which is the second most prominent PUFA in the skin and is involved in many inflammatory reactions [22]. In our study, both LA and AA showed distributions in the vermilion as well as in the skin, suggesting that these two PUFAs play important role(s) in both regions of the lip. 

In subject 1 and 2, POA and OA showed a distinct distribution pattern in the lower area of the skin, the area rich in various secretory glands, suggesting that the constituents and/or secretions of the glands may affect their distributions. These two MUFAs may have a specific role (e.g., lubrication) in the skin. Previously, topical application of POA was found to improve wound healing through anti-inflammatory activity in rats [34], and OA was reported to play a role in wound closure [35]. However, an inter-individual variability of the distributions of these two MUFAs was observed when compared with that of subject 3.

Fatty acids have emerged as a potential pharmaceutical ingredient in the treatment of skin diseases due to their multiple biological activities including anti-inflammatory and antioxidant properties [36]. Knowledge of the distribution of these endogenous compounds is important for developing a biocompatible drug for a target organ. Our study revealed the specific and differential distributions of several fatty acids in the vermilion as well as in the skin of the human lip and can thus provide a novel therapeutic approach for the treatment of lip disorders. We believe that our data are informative in obtaining an understanding of the role of FFA in the function of human lip tissue.

However, this study has some limitations. We were unable to find the distribution pattern of FFAs in the oral mucosa in comparison with skin and vermilion due to the scarcity of the oral mucosa tissue within the samples and the limited number of samples. An increase in the number of samples and inclusion of oral mucosa tissue within the samples are required for more comprehensive studies to explore the functional properties of DHA specific to the vermilion of the human lip. Additionally, it would be important to analyze if FFAs affect the distributions and quality of membrane lipids in the vermilion and skin.

## 4. Materials and Methods

### 4.1. Tissue Sample

The protocol for obtaining human lip tissue samples was approved by the ethical committee of Niigata University (project identification code: 2015–5018; June 26, 2013), and this study was also approved by the ethical committee of the Hamamatsu University School of Medicine on the 26th of September 2018 (Code: 18-153). Patients who were undergoing cheiloplasty at the participating hospitals were provided with sufficient information, and the guardians of all participating individuals signed informed consent forms. Lip specimens (*n* = 3) were obtained by trimming off during cheiloplasty. Immediately after dissection, the tissue was embedded in 2% (*w/v*) carboxymethyl cellulose solution, placed in a tube, frozen on dry ice, and transported to the laboratory. The tissue was then stored at −80 °C until analysis.

### 4.2. Sample Preparation for DESI–MSI Analysis

The tissues were sagittally sliced to a thickness of 10 µm using a cryostat (CM1950; Leica, Wetzler, Germany) at −20 °C and mounted on glass slides (Matsunami, Osaka, Japan).

### 4.3. DESI–MSI Analysis

MSI analysis was performed using a desorption electrospray ionization (DESI) source attached to a quadrupole time-of-flight (Q-TOF) mass spectrometer (Xevo G2-XS Q-TOF, Waters, Milford, MA, USA) in negative ion mode. A software-controlled 2D moving stage was used to scan the defined area with a scan rate and pixel size of 200 µm/sec and 100 µm × 100 µm, respectively. The spray solvent (98:2 methanol/water, *v*/*v*) was delivered at a flow rate of 2 µL/min using a solvent pump (ACQUITY UPLC Binary Solvent Manager, Waters, Milford, MA, USA). Mass resolution, mass window, and collision energy were set at 20000, 0.02 Da, and 4.00 V, respectively. The DESI source conditions were optimized as a capillary voltage of 4.0 kV, a nebulizing nitrogen gas pressure of 0.4 MPa, an ion transfer capillary (mass inlet) temperature of 120 °C, a spray impact angle of 70°, and a sampling cone of 50. Analyzer mode and data type were set as “sensitivity” and ”continuum”, respectively. Mass spectra of the 1000 highest-intensity peaks were collected in a mass range of *m*/*z* 50 to 400. The emitter was exposed to the sprayer tip by approximately 0.5 mm. The emitter tip-to-surface, emitter tip-to-mass inlet, and mass inlet-to-surface distances were approximately 2, 6, and 0.5 mm, respectively. Prior to the measurements, the mass spectra were calibrated externally using sodium formate solution (500 µM) in 2-propanol: water (90:10, *v*/*v*), and the detector setup was performed using leucine enkephalin solution (500 µM). At least three serial sections for each individual subject were analyzed under the same experimental conditions to assess the reproducibility of the spectra. After imaging, the tissue sections were subjected to hematoxylin and eosin (H&E) staining for histological analysis.

For mass accuracy corrections, we used lock mass correction. An abundant ion of *m*/*z* 281.2486 (exact *m/z* of oleic acid) was used as mass lock with a tolerance and minimum signal intensity of 0.05 amu and 10 counts, respectively.

### 4.4. Data Analysis

In HDImaging software (Waters, Milford, MA, USA), spectra were normalized using total ion current (TIC), and 2D ion images were constructed to visualize the spatial distributions of the detected molecules from the 1000 most intense peaks of each mass spectra. Normalized spectra were exported to the MassLynx V4.1 software (Waters, Milford, MA, USA), wherein the intensities were further normalized to the largest peak on display.

The assignments of the fatty acids were based on the *m/z* values and their mass tolerances, previous lipid studies using DESI–MSI [16,17,20,21], as well as The Human Metabolome Database (http://www.hmdb.ca/spectra/ms/search) [37].

We defined the skin, epithelium of vermilion, and its underlying tissue areas on the ion image with reference to the H&E staining of the same section. To observe the region-specific distribution of the molecules, H&E images were overlaid on the ion images.

## 5. Conclusions

In conclusion, our results show that the FFA distributions are different in vermilion and skin. Among the five FFAs detected in our study, DHA is highly distributed in the vermilion and can thereby distinguish the vermilion from the skin. Our findings can provide a new therapeutic approach in the treatment of lip disorders.

## Figures and Tables

**Table 1:** Demographic data of the subjects in this study.

Subject ID	Age	Sex	Diagnosis	Sample Collection Period
1	6 months	Female	Cleft lip, alveolus and palate, right side	April, 2018
2	8 months	Male	Cleft lip, alveolus and palate, right side	March, 2019
3	5 months	Female	Cleft lip and alveolus, Left side	May, 2019

**Table 2:** List of assigned peaks in the spectra of the human lip obtained by DESI–MSI in negative ion mode.

*m*/*z* Observed	Monoisotopic Mass (M-H)^-^	Molecular Assignments	Mass Error (Δppm)	Reference(s)
253.2171	253.2173	Palmitoleic acid(C16:1)	0.8	[17]
279.2328	279.2330	Linoleic acid (C18:2)	0.7	[17,20]
281.2485	281.2486	Oleic acid (C18:1)	0.4	[16,17,20,21]
303.2329	303.2330	Arachidonic acid (C20:4)	0.3	[16,20,21]
327.2328	327.2330	Docosahexaenoic acid (C22:6)	0.6	[16,20]

## Supplementary Materials

Supplementary materials can be found at https://www.mdpi.com/1422-0067/21/8/2807/s1.
