# Supplementary material for: Higher Accumulation of Docosahexaenoic Acid in the Vermilion of the Human Lip than in the Skin
Source: Int J Mol Sci. 2020 Apr 17;21(8):2807. doi: 10.3390/ijms21082807 (PMC7215545; doi:10.3390/ijms21082807)
Supplement: Supplementary file 1 [file ijms-21-02807-s001.pptx]

## Slide 1
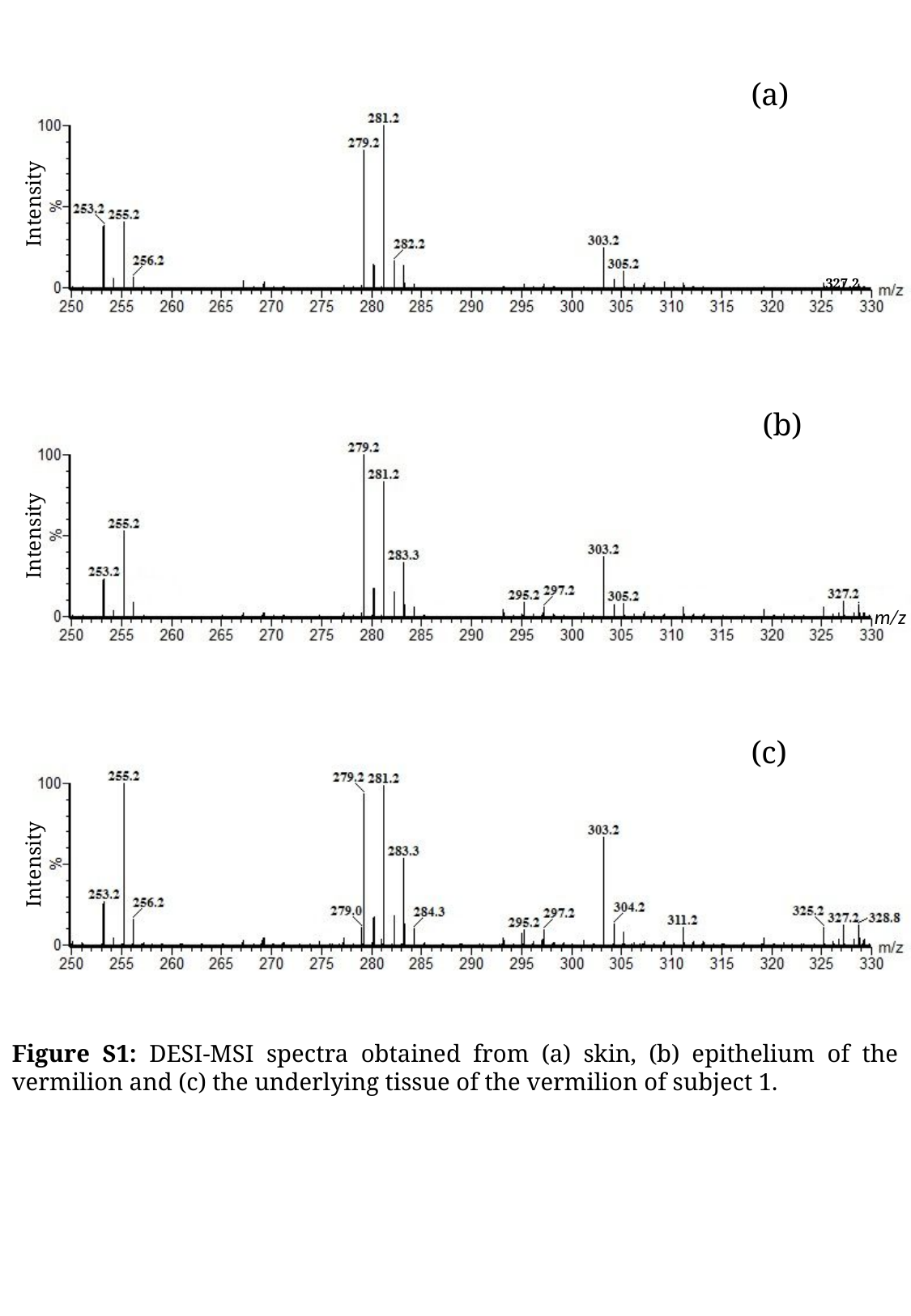

(a)
Intensity
327.2
(b)
Intensity
m/z
(c)
Intensity
Figure S1: DESI-MSI spectra obtained from (a) skin, (b) epithelium of the vermilion and (c) the underlying tissue of the vermilion of subject 1.
